# The Process of Pasting and Gelling Modified Potato Starch with LF-NMR

**DOI:** 10.3390/polym14010184

**Published:** 2022-01-03

**Authors:** Katarzyna Walkowiak, Krzysztof Przybył, Hanna Maria Baranowska, Krzysztof Koszela, Łukasz Masewicz, Michał Piątek

**Affiliations:** 1 Department of Physics and Biophysics, Poznań University of Life Sciences, Wojska Polskiego 38/42, 60-637 Poznan, Poland; katarzyna.walkowiak@up.poznan.pl (K.W.); hanna.baranowska@up.poznan.pl (H.M.B.); lukasz.masewicz@up.poznan.pl (Ł.M.); 2 Department of Dairy and Process Engineering, Food Sciences and Nutrition, Poznań University of Life Sciences, Wojska Polskiego 31, 60-624 Poznan, Poland; krzysztof.przybyl@up.poznan.pl; 3 Department of Biosystems Engineering, Poznań University of Life Sciences, Wojska Polskiego 50, 60-625 Poznan, Poland; 4 Department of Meat Technology, Food Sciences and Nutrition, Poznań University of Life Sciences, Wojska Polskiego 50, 60-625 Poznan, Poland; piatek@up.poznan.pl

**Keywords:** LF-NMR, modified potato starches, pasting, starch gels, microwave modifications

## Abstract

Currently, society expects convenience food, which is healthy, safe, and easy to prepare and eat in all conditions. On account of the increasing popularity of modified potato starch in food industry and its increasing scope of use, this study focused on improving the physical modification of native starch with temperature changes. As a result, it was found that the suggested method of starch modification with the use of microwave power of 150 W/h had an impact on the change in starch granules. The LF-NMR method determined the whole range of temperatures in which the creation of a starch polymer network occurs. Therefore, the applied LF-NMR technique is a highly promising, noninvasive physical method, which allows obtaining a better-quality structure of potato starch gels.

## 1. Introduction

In recent years, one can observe an increased interest in modified starch (starch derivatives). As a natural polysaccharide (biopolymer), starch is [1] one of the most frequently used products in the food industry. This results from the fact that the above biopolymer is easily accessible as a main ingredient in potatoes [2] and grains.

Taking into consideration the biodegradability of starch [3], for many years, one can observe numerous attempts to modify starch of various botanical origins in order to widen the range of use of this material, as well as to change its properties. In the textile industry, starch is used as a binding material in the production of corrugated cardboard [4,5]. In the food industry, taking into account its natural properties, starch is used to produce gels [6,7], thickening agents [1,8], and carriers in spray-drying of concentrated fruit juices and as starch syrup [9,10]. Starch and its derivatives are said to be good carriers in spray-drying. They are characterized by high molecular weight [10,11] and high glass transition temperature; unfortunately, starches are characterized by a low ability to create membranes [12].

The most noticeable modification of starch is chemical modification, which introduces addition functional groups into the system and is met with numerous restrictions as a food additive. However, in the pharmaceutical industry, starch derivatives are versatile compounds, which are commonly used to design systems of delivering medicines [13]. One of the least invasive methods of modifying starch grains, which is also popular in the specialized literature, is physical modification [1]. This process is based on affecting starch grains with electromagnetic radiation in the microwave range [14,15,16,17]. Unfortunately, the radiation of starch with electromagnetic waves has a negative impact on its properties [18]. Currently, consumers are seeking healthy and safe food, as well as the use of those products in various food forms. That is why it is important to establish what influence the modifying technique of an element of potato starch can have on the quality condition of food. In view of the above, emphasis is put on searching for noninvasive modifying techniques, which above all do not impact the chemical composition of the product that is used. Compared with other vegetable starches, potato starch demonstrates the highest maximum stickiness and surprisingly highly transparent properties of gruel. Thanks to those properties, it is widely used in the food industry. That is why it is vital to analyze the changes in the gelling properties of modified potato starch [19,20].

It is worth noting that low-field nuclear magnetic resonance (LF-NMR) was used for the needs of this research. Currently, it is one of the most modern techniques of determining surface relaxation in products [21] during the process of food modification. This method does not use noxious radiation (for example, X-ray radiation), which could have a negative impact on the product under research. The specialist literature describes the nuclear magnetic resonance method (NMR) as noninvasive and accurate, which does not have any impact on the physical properties of the product [22,23,24,25]. By comparing the LF-NMR method used in temperature research with literature data using a Brabender viscograph (PN-A-74706:1984) [26], e.g., RVA (Rapid Visco Analyzer), once can determine the temperature range in which the process of gel structure formation occurs [27]. When conducting rheological research, one should provide the temperature of pasting at the point with the highest stickiness value or maximum temperature reached in the system [28,29].

Accordingly, an endeavor was made to analyze the process of pasting of modified materials with a low-field NMR spectrometer, which works well with determining the dependencies of relaxation times, which in turn pictures the movement of water molecules in the system under research [30,31,32]. The LF-NMR technique is widely used to research food products such as bread [33,34] or cheese [35]; it also allows a thorough analysis of molecular dynamics [36].

The aim of this paper was the physical modification of native starch with temperature changes, as well as the evaluation of influence of the above modification on the process of pasting and creating gel structures in the samples that were subject to analysis. It should be noted that the research was supported by the innovative LF-NMR technique.

## 2. Materials and Methods

### 2.1. Potato Starch

Potato starch, type Superior Standard (PPZ Trzemeszno), was used for the needs of the research. It should be added that the inlet humidity was set to 35%. Samples of native starch were prepared, which were closed in containers in order to carry out the process of physical modification with a microwave vacuum dryer according to the methods described by Zhang [37].

### 2.2. Microwave Modification

**Figure 1:**
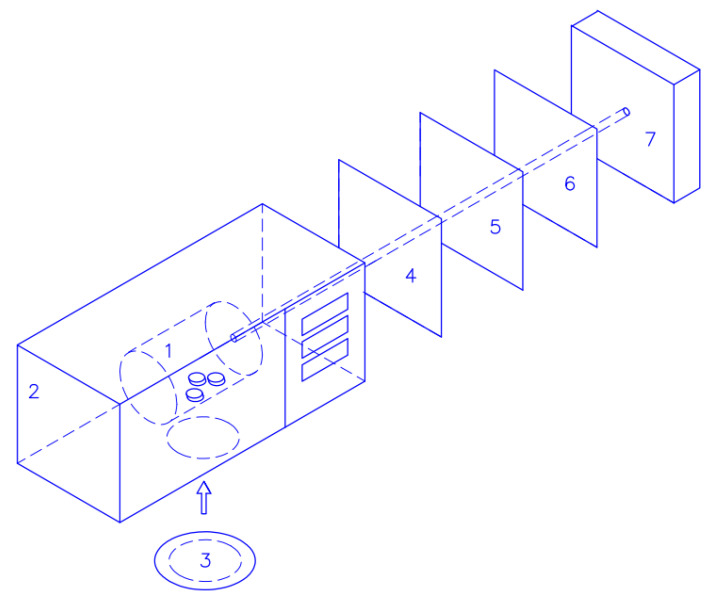
Diagram of microwave vacuum drying apparatus: 1—vacuum drying drum, 2—microwave chamber, 3—magnetron, 4—drum drive, 5—condenser, 6—manometer, 7—vacuum pump.

The research on modifying native starch was carried out using a microwave vacuum dryer Promis Tech. Device as a radiator that uses a magnetron of 2.45 GHz and reaches vacuum at 30–40 kPa. The samples were placed inside the rotary vacuum drum (1), located inside the microwave chamber (2) (Figure 1). Inside the drum there is a possibility to regulate air pressure, with an estimated 3% of atmospheric pressure (30 hPa). The product can change its location (can move) in the rotary drum; hence, one can avoid the possibility of local overheating of sample. As a result, the rate of radiation is evenly distributed across the whole volume [38,39,40]. In order to prepare modified starch, a specific microwave radiation power (50 and 150 W/g) was used, and the time of modification was between 1 and 4 min. The measurement of the flow velocity of the drying agent was controlled by means of an anemometer.

### 2.3. Biopolymer System

The obtained samples were characterized by various degrees of modification and were later used to prepare starch suspensions, which, after heating, became 5% gels. Next, solutions were prepared (10 mL each) consisting of 0.5 g of starch supplemented with distilled water, for both native and modified starch. Suspensions that were obtained were mixed with magnetic mixer in the form of magnets fixed to the work surface. After mixing, all suspensions were left for 24 h. The samples were stored for 24 h in room conditions in order to obtain gruels at room temperature (the temperature of gruels was identical to the temperature inside the room). The above allowed obtaining a stable polymer structure in the samples under research. During that time, the systems were not subject to long-term processes of the retrogradation of amylopectin chains [41].

### 2.4. Temperature Research

In order to determine the temperature changes of spin–network *T*_1_ and spin–spin *T*_2_ relaxation times, an impulsive spectrometer H NMR (Ellab Poznań, Poland) was used. It should be added that the above device operates at the frequency of 15 MHz and includes an integrated system of temperature control. In order to measure spin–network relaxation times, a shifted impulse sequence and roots were used [42]. Distances between impulses (*T*_1_) were changed from 100 to 6500 ms. The repetition time TR was 20 s, and a single sequence consisted of 32 repetitions. A total of 119 measurement points from each of the 32 FID signals were collected in order to carry out the calculation. Measurements of spin–spin relaxation times were carried out with Carr–Purcell–Meiboom–Gill (CPMG) impulses [42,43,44,45,46]. Distances between impulses ranged from 10 to 15 ms. Measurements of both relaxation times were carried out in the 20–90 °C temperature range. It is worth adding that each sample was both heated and cooled.

## 3. Results and Discussion

**Figure 2:**
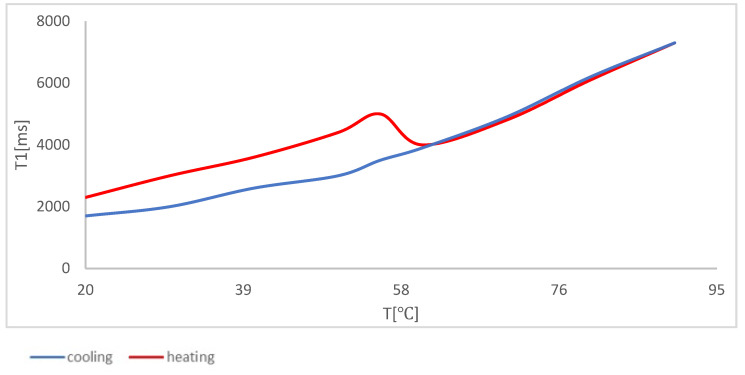
Temperature dependency of spin–network relaxation time for native potato starch.

The research was conducted in a wide range of temperatures, which allowed determining the mechanisms of influence at the molecular level. Research on the temperature of starch suspension reflects the influence of the physical modification of starch on the process of pasting and gelling in the samples that were analyzed. It is worth noting that there was a correlation between relaxation time *T*_1_ and temperature, which is a classical feature when creating gels from a suspension (Figure 2). This process can be divided into three areas. When heating the suspension of potato starch, one can observe an increase in relaxation time together with an increase in temperature, which is the effect of delivering energy to the system. Next, one can observe the area characterized by refraction related to the formation of a polymer network structure. In macroscopic research, for example, stickiness is recorded only at the highest temperature, which reflects the temperature of pasting. The LF-NMR method allows determining the whole temperature range in which network units are formed. The last area (for temperatures in the range of 60 °C to 90 °C) shows an increase in relaxation time together with a temperature increase, as in the case of the first area; however, it is related to sol and not to suspension. During the process of cooling down samples, one can observe a monotonous decrease in relaxation times together with a temperature decrease, which proves that the starch was completely pasted. In this area, one can observe a transition of sol to gel.

During the analysis of temperature dependencies of spin–network relaxation times of modified potato starch (during the process of heating up the samples), a clear decrease in *T*_1_ times in comparison with the sample of native starch was observed. Regardless of the type of modification, all samples that were subject to the analysis of temperature changes were characterized by a decrease in *T*_1_ time, which means that starch exposed to electromagnetic waves exhibits a faster reorganization of water molecules than before modification. Samples which were subject to physical modifications definitely changed their final moisture (Table 1).

**Figure 3:**
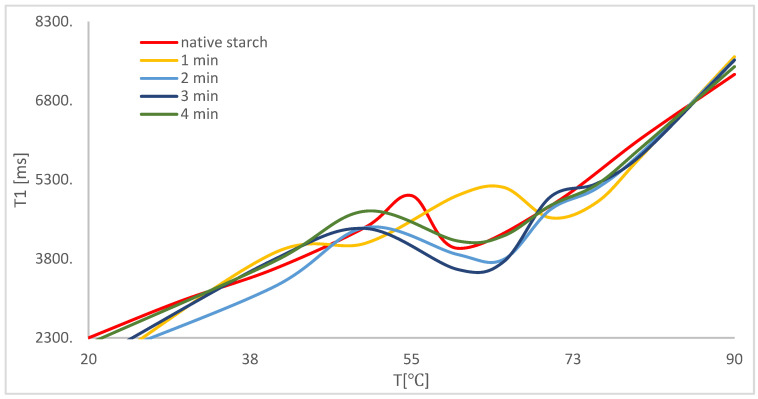
Temperature correlation of spin–network relaxation time during the process of heating up samples after modification with 50 W/g.

**Figure 4:**
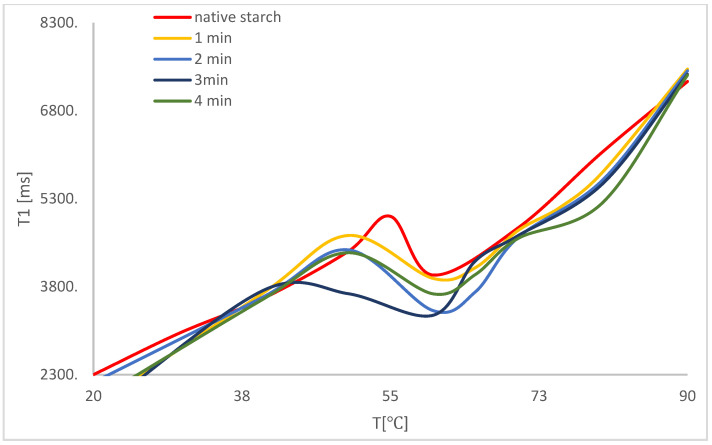
Temperature correlation of spin–network relaxation time during the process of heating up samples after modification with 150 W/g.

Demand for hydration water, which is a cause of water shortage in samples, influences the process of expansion and the initial stage of pasting starch granules. For native starch, the initial temperature of pasting is in the 50–60 °C range. Modification with microwaves influenced a decrease in the temperature range. In the case of modification with 50 W/g (Figure 3) and 150 W/g (Figure 4) independently of modification time, the initial temperature of starch pasting and the whole process became more scattered over time.

Modification with 50 W/g within the period of 1 min (Figure 3, yellow line) led to changes in pasting temperature by 10 °C, while resulting in the largest changes in the last area of temperature changes, in which one can observe another temperature peak of 65 °C. It should be added that this phenomenon does not occur in the case of native starch. Moreover, one can observe a maximum in all samples under modification, which indicates an influence on changes in the structure of starch grains.

Despite physical modification by microwave treatment [47,48,49,50], one can observe a further refraction area, which is typical of potato starch, as well as a minimum period of spin–network relaxation time. The above means that, during the process of polymer network formation, one can observe water bound in starch grains. Modification with 150 W/g within a period of 1 min led to a substantial decrease in the value of moisture content, connected to the change in affinity between the interaction of starch and water. The above is related to changes from a hydrophilic effect to a hydrophobic effect. The reason for the move may be the change in crystal polymer structure.

Additional curve refraction suggests that some water is trapped in crystal structure type A when potato starch granules are subject to the effect of electromagnetic activity. It should be added that changes in the crystal structure in potato starch granules from type B to type A were previously observed [14]. The results explain the molecular mechanism of changes in crystal structure. Energy from electromagnetic waves is absorbed by water molecules situated inside the structure, causing a reorganization of water–polymer bonds and resulting in them being closer inside the structure. The higher temperature, for which other conformational transitions of polymer were observed, is related to the release of those water molecules trapped inside the crystallite.

Upon analyzing the process of temperature changes, one can observe that the type of modification which shortened the temperature of pasting process to the largest extent also led to an increase in maximum in the last area of heating up samples. One can also conclude that the process of polymer network structure formation was also the longest.

**Figure 5:**
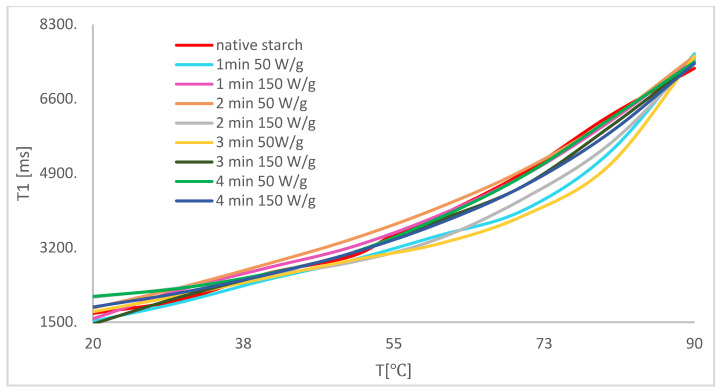
Temperature correlation of spin–network relaxation time during the process of cooling the samples.

Physical modification by microwave treatment of potato starch [17,50] does not change the course of temperature correlations during the process of cooling down the samples. Irrespective of the type of modification, one cannot observe any significant changes in temperature analysis, which means that all structures that were formed underwent complete transformation from sol to gel (Figure 5).

LF-NMR research showed that physical modification by microwave treatment of starch has an influence on the faster reorganization of water molecules, which leads to gel structure formation. Other studies on physical modification by microwave treatment of potato starch proved that such a modification leads to starch becoming an emulsion stabilizer and emulsifier or fat substitute [51]. It should also be emphasized that the research determined the shift in temperature range responsible for structure formation and starch pasting in the direction of lower temperatures compared with native starch. On the basis of research progress related to problems regarding starch modification, it can be clearly stated that the technique applied is both innovative and environmentally friendly. It was proven in the literature that modification with the use of microwaves in order to obtain starch does not have a negative impact on the environment and consumer health [50]. On account of weak starch stability, endeavors have been made to strengthen its specific functional properties [52] by promoting various methods of modification including microwaves.

## 4. Conclusions

The results of this study allow concluding that starch changed its properties as a result of the effect of the electromagnetic field. The use of electromagnetic radiation in starch with 150 W/g led to changes in the structure of starch granules, which permanently changed the hydrophobic and hydrophilic properties. The length of polymer network affected by temperature changes is one of the structural parameters of starch with a fundamental influence on physiochemical properties. Low-field NMR method allows determining the whole temperature range in which the polymer network structure formation of starch occurs. Research with physical factors allows obtaining potato starch modifiers, which, at lower temperatures of heating up, undergo pasting and form a gel structure. This information can be very significant when using starch as a gelling factor in food products, as well as technologically, because one needs a lower temperature when preparing paste.

## Figures and Tables

**Table 1:** Parameters of physical modification by microwave treatment and final moisture of starch.

Name	Modification Time (min)	Power (W/g)	Moisture (%)
Native starch	-	-	34.48
1	1	50	35.02
2		150	22.41
3	2	50	21.69
4		150	5.99
5	3	50	21.53
6		150	7.35
7	4	50	14.19
8		150	4.21

## Data Availability

Not applicable.
